# Development of a survey evaluating patient Concerns Over Peri‐operative Events (COPE)

**DOI:** 10.1002/anr3.70098

**Published:** 2026-09-02

**Authors:** A. J. Gardner, T. Potter, P. Greig, G. Radcliffe, J. E. O'Carroll, K. El‐Boghdadly

**Affiliations:** ^1^ Department of Anaesthesia and Peri‐operative Medicine Guy's and St Thomas' Hospital London UK; ^2^ Centre for Human and Applied Physiological Sciences King's College London London UK

**Keywords:** anxiety, patient reported outcome measures, pre‐operative care, surveys and questionnaires, validation study

## Abstract

Anxiety frequently burdens patients before surgery, potentially leading to poorer recovery. However, standard assessment tools typically quantify the intensity of this distress without understanding its root cause. This often leaves clinicians unaware of the specific fears driving a patient's anxiety. We aimed to address this deficit by developing and validating a questionnaire designed to identify individual pre‐operative concerns. An iterative development process resulted in a 14‐item questionnaire: Concerns Over Peri‐operative Events (COPE). We conducted a prospective evaluation of 52 patients having elective surgery, measuring the questionnaire's performance against the Amsterdam Pre‐operative Anxiety and Information Scale. The questionnaire performed well on metrics of validity, reliability and acceptability. Severe pain was the most reported concern. The median (IQR [range]) total COPE score was 30 (22‐44 [14‐63]). There was a positive correlation between the Amsterdam Pre‐operative Anxiety and Information Scale and COPE scores. A classification model to predict Amsterdam Pre‐operative Anxiety and Information Scale‐positive status demonstrated excellent discrimination with an area under the curve of 0.86. An optimal COPE score of ≥ 34 demonstrated 88% sensitivity and 81% specificity. While future research should focus on generalisability, this novel questionnaire of patient concerns facilitates the identification of specific drivers of pre‐operative anxiety. By bridging the gap between clinicians' assumptions and patient experience, the COPE questionnaire may facilitate targeted therapies and improve informed consent.

## Introduction

Between 33 and 90% of patients report anxiety during their peri‐operative care [1, 2, 3, 4, 5]. This can be described as a subjective state of emotional unease ranging from mild apprehension to severe anxiety with autonomic dysfunction relating to anaesthesia or the surgery itself [4]. The first UK Sprint National Anaesthesia Project (SNAP‐1) reported that anxiety was most frequently described as the worst aspect of patients' anaesthetic experience [6]. Pre‐operative anxiety may be associated with adverse postoperative outcomes, including increased incidence of postoperative nausea and vomiting, higher analgesic requirements and an increased risk of chronic post‐surgical pain [7, 8]. These complications might have long‐term implications, can be associated with poor patient experience and may result in future aversion to anaesthesia and surgery [9].

Pre‐operative anxiety may be caused by negative beliefs or concerns regarding anaesthesia or surgery. Common causes of pre‐operative anxiety include postoperative pain, accidental awareness during general anaesthesia and death [1, 2, 3, 4, 10, 11]. It is generally difficult to accurately assess and thus manage patient anxiety in the pre‐operative period [12]. Existing methods of measuring pre‐operative anxiety include the Spielberger State‐Trait Anxiety Inventory [13] and the Amsterdam Pre‐operative Anxiety and Information Scale (APAIS) [14]. The APAIS is a self‐reported questionnaire administered on the day of surgery, consisting of six items that measure both the patient's desire for more information and their anxiety levels. It has been validated in various patient populations and languages since its inception by Moerman et al. in 1996 [15, 16, 17]. While these established tools can be used to measure anxiety pre‐operatively, they do not attempt to identify the underlying specific cause of anxiety.

Concerns described by patients may differ from those published in the literature and those commonly assumed by anaesthetists during the pre‐operative assessment and consent process [18]. There is a need to align clinicians' understanding of important concerns with that of patients [1]. Therefore, a tool to identify specific concerns causing an individual's anxiety might allow early identification and intervention to manage concerns and improve patient experience and outcomes [13].

The primary aim of this study was to develop a questionnaire which elicits patient concerns before elective surgery. The secondary aim was to assess reliability, acceptability and feasibility of the questionnaire and correlate it with the patients' state of anxiety.

## Methods

We aimed to develop a questionnaire for potential use in future care. Following review by local Research and Development tools, as well as confirmation by the National Health Service Health Research Authority Policy framework decision aid, it was deemed a service evaluation [19]. The project was prospectively approved as a service evaluation. Informed consent was sought from patients.

The survey was designed iteratively. Firstly, potential question items for the Concerns Over Peri‐operative Events (COPE) questionnaire were sourced from a directed review of the literature to produce a comprehensive list of patient concerns [1, 2, 3, 4, 12, 20, 21, 22]. The list was subjected to thematic analysis, grouping related concerns. A multidisciplinary panel with expertise in anaesthesia, surgery, critical care, peri‐operative medicine, psychology and psychiatry provided feedback on the relevance and intelligibility of the questions. A patient and public involvement group was consulted, and seven patients underwent semi‐structured interviews at pre‐operative assessment to ensure the intelligibility, usability and acceptability of all questions.

The initial survey format was given to 82 patients. Based on feedback from this initial pilot, self‐reporting of anxiety was replaced with the validated APAIS, presented using five‐point Likert scales as found elsewhere in our survey. As per the original Moerman et al. study, an APAIS anxiety score of ≥11 was used to define the presence of pre‐operative anxiety and ≥5 for a patient's requirement for more information [14]. The survey items were also rationalised, guided by interitem correlations and expert advice. For example, the question which correlated most with other item responses (fear of an ICU stay, mean r^2^ = 0.61) was removed. Another two questions with similar concepts (responsibilities affected at home, delays getting back to work) were merged (r^2^ = 0.78). However, some questions remained despite either low correlation or low concern rating, because of relevance to existing peri‐operative consent practices (damage to teeth, major adverse surgical complications).

Patients' questionnaire scores were classified against a dichotomous APAIS anxiety status defined above [14]. An optimal score threshold was defined based on sensitivity and specificity.

The survey included questions to capture baseline patient demographics, current and past medical, anaesthetic and surgical history. Surgical specialty, operation and planned anaesthesia type were recorded. The planned surgical operation was stratified by complexity as minor, intermediate, major or major complex [23]. We analysed whether patients' questionnaire scores and APAIS status were associated with demographic or clinical factors.

Eligible participants were aged 18 years or older, undergoing elective surgery and able to complete the survey in English. The survey was completed less than 24 hours before surgery. For validation of the survey, a convenience sample was used due to the variation in patient numbers scheduled for elective surgery each day.

We compared the COPE score with the APAIS and measured interitem correlations to assess convergent validity. Construct validity was assessed by comparing COPE scores between patient gender, surgical type and previous ASA physical status. Sampling adequacy was assessed with Kaiser‐Meyer‐Olkin and Bartlett's Test of Sphericity prior to factor analysis [24]. Discriminant validity was assessed by correlating patients' responses to the COPE questionnaire with the APAIS score. Internal consistency was measured using Cronbach's α as an average correlation between each item of the survey, and split‐half reliability was measured with the Guttman Split‐Half Coefficient. Feasibility and acceptability were assessed by recording the time taken for patients to complete the questionnaire and the successful completion and return rates. Readability of the questionnaire was assessed using Microsoft Word (Microsoft, Inc. Redmond, WA) to generate the reading age required to understand the document.

Data are presented as either mean (SD) or median (IQR [range]), with 95%CI presented as appropriate. Normality was assessed using the Kolmogorov–Smirnov test. Spearman's Rank correlation was used for bivariate correlations, and internal consistency with Cronbach's α. Ordinal data of self‐reported health and the ASA physical status classification system were divided to generate dichotomous variables of approximately equal group sizes. For classification analyses, a receiver operating characteristic (ROC) curve was used, comparing the continuous COPE score with the dichotomous APAIS anxiety status as a classifier. From these, coordinate points determined the relative specificity and sensitivity at a given threshold. For categorical analyses between the COPE score and APAIS, the two‐sided Fisher's exact test was used to account for low cell counts. For subgroup analysis, patients were dichotomised by age, gender, ASA physical status, day case status, mental health history and self‐reported health rating. Actual p values are reported unless under 0.001. Statistical analyses were performed using SPSS for Windows v26.0 (IBM Corp., Chicago IL. USA). Any incomplete questionnaires were excluded from data analysis.

## Results

The iterative process ensured the final COPE questionnaire maintained an optimal balance between data collection, intelligibility and practicality (Supporting Information Appendix S1). The final COPE questionnaire consisted of 14 questions regarding patient concerns. Responses were collected using five‐point Likert scales and then summated to provide a total COPE score with a maximum of 70 points. Within the semi‐structured interviews, the survey took a median 6 min (4.5‐7.0 [4‐11]) for patients to complete. An assessment of readability yielded a Flesch Reading Ease Score of 57.6 and a Flesch–Kincaid Grade level of 7.7, corresponding to a UK reading age of 12–13 years of age.

**Figure 1:**
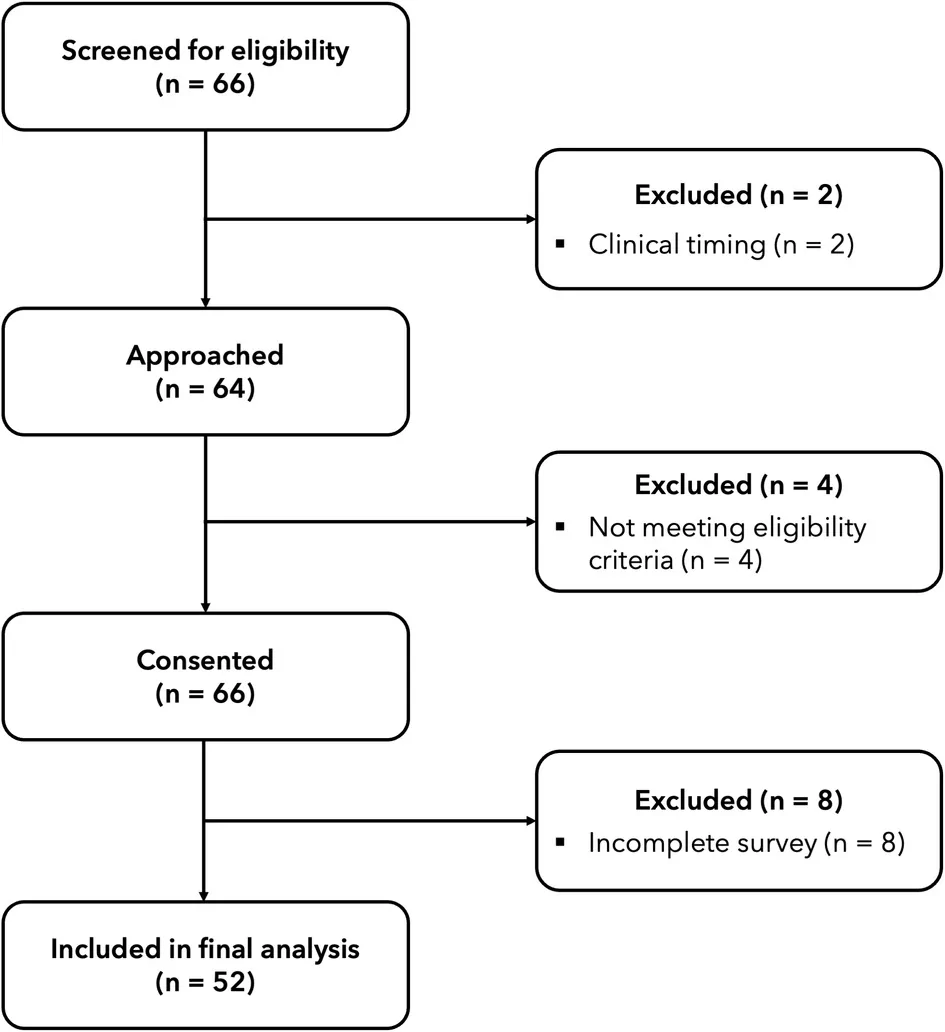
Flowchart of participant selection and inclusion.

Of the 66 patients screened, 52 were included in the final analysis (Figure 1). The patients' characteristics are presented in Table 1. Operative characteristics are summarised in Supporting Information Appendix S2.

**Table 1:** The demographic and medical characteristics of the patients, along with their Amsterdam Pre‐operative Anxiety and Information Scale responses (n = 52). Categorical variables rounded to integers, continuous variables to 1 decimal place. GCSE, General Certificate of Secondary Education.

Variable	Value
**Age; years**	58.6 (16.0)
**Gender; male**	29 (56%)
**Ethnicity**	
White	45 (87%)
Black	4 (8%)
Asian	1 (2%)
Arab	1 (2%)
Mixed	0 (0%)
No data	1 (2%)
**Education**	
University/Higher education	20 (38%)
GCSE/None	15 (29%)
A‐Level	11 (21%)
Prefer not to say	4 (8%)
No data	2 (4%)
**Marital status**	
Single	7 (13%)
Married, partnered, civil partnered	33 (63%)
Widowed, divorced, separated	12 (23%)
No data	‐
**Employment**	
Employed, self‐employed, carer	28 (54%)
Unemployed, retired, student, volunteer, unfit to work	23 (44%)
No data	1 (2%)
**Dependants**	
Yes	15 (29%)
No	36 (69%)
No data	1 (2%)
**Health self‐rating**	
Very good	8 (15%)
Good	21 (40%)
Fair	18 (35%)
Bad	3 (6%)
Very bad	1 (2%)
No data	1 (2%)
**Mental health history**	
Anxiety	12 (23%)
Depression	11 (21%)
Other	3 (6%)
None	30 (58%)
No data	5 (10%)
**ASA physical status**	
1	4 (8%)
2	23 (44%)
3	16 (31%)
4	9 (17%)
**Previous general anaesthetic**	
Yes	43 (83%)
No	9 (17%)
**Previous neuraxial anaesthetic**	
Yes	15 (29%)
No	33 (63%)
No data, not sure	4 (8%)
**Seen an anaesthetic doctor before survey**	
Yes—during hospital stay	2 (4%)
Yes—during pre‐assessment clinic	15 (29%)
No	33 (63%)
No data	2 (4%)
**Anaesthetic explained?**	
Yes	24 (46%)
No	16 (31%)
Not sure	5 (10%)
No data	7 (13%)
**Amsterdam Pre‐operative Anxiety and Information Scale (APAIS)**	
Mean anxiety score (4–20)	9.0 (3.6)
Positive anxiety status (≥11)	31%
Mean information requirement score (2–10)	5.4 (2.4)
Positive information requirement status (≥5)	62%

The survey demonstrated no obvious floor or ceiling effects; respondents used the full lengths of the scales available, and no more than 15% of subjects scored the minimum or maximum scores [25]. The Kaiser‐Meyer‐Olkin test result of 0.845 suggested sampling adequacy; Bartlett's test for sphericity was <0.001. Cronbach's α was 0.93.

**Figure 2:**
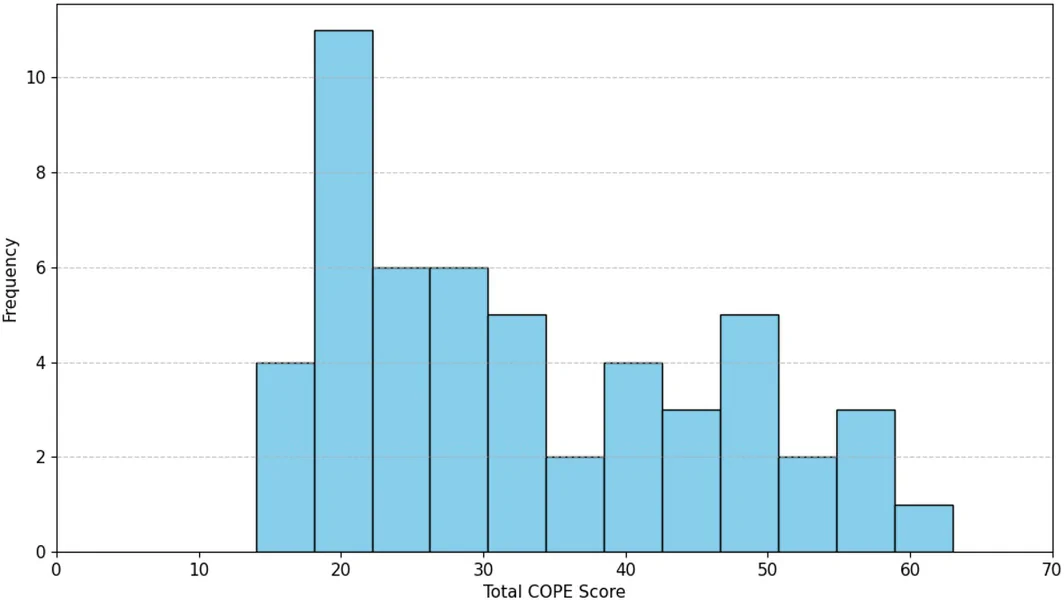
A histogram of final Concerns Over Peri‐operative Events (COPE) questionnaire scores.

The median (IQR [range]) COPE score was 30 (22‐44 [14–63]) out of a maximum of 70. COPE scores (Figure 2) were log_10_‐transformed to normalise the variable. The transformed COPE score was therefore used in further analyses.

Pain was the most reported concern, followed by major operative complications and then an unsuccessful operation. Embarrassment and dental damage were scored lowest (Table 2).

**Table 2:** The mean per‐item Concerns Over Peri‐operative Events (COPE) questionnaire responses, with the standard deviation (SD); rounded to 1 decimal places.

Item	Concern	Mean (SD)
Q1	Accidental dental damage	1.6 (1.1)
Q2	A sore throat postoperatively	1.7 (1.0)
Q3	Postoperative nausea and vomiting	2.4 (1.2)
Q4	Severe pain postoperatively	3.0 (1.3)
Q5	Unintentional operative awareness	2.4 (1.5)
Q6	Not waking up postoperatively	2.6 (1.7)
Q7	Permanent scarring or deformity	2.1 (1.3)
Q8	An unsuccessful operation	2.8 (1.5)
Q9	The wrong operation performed	2.0 (1.5)
Q10	Embarrassment whilst asleep	1.6 (1.1)
Q11	Normal activities affected	2.5 (1.4)
Q12	A minor complication	2.7 (1.3)
Q13	A major complication	2.9 (1.6)
Q14	The risk of dying	2.7 (1.6)

The mean APAIS anxiety score was 9.0 (3.6) and APAIS information requirement score 5.4 (2.4). Therefore, 16 (31%) had a positive anxiety score and 32 (62%) a positive attitude to receiving more information.

There was a positive correlation between participant APAIS and COPE scores, Spearman's ρ = 0.686 (p < 0.001). There were no significant associations between the COPE and any of the prespecified subgroups.

**Figure 3:**
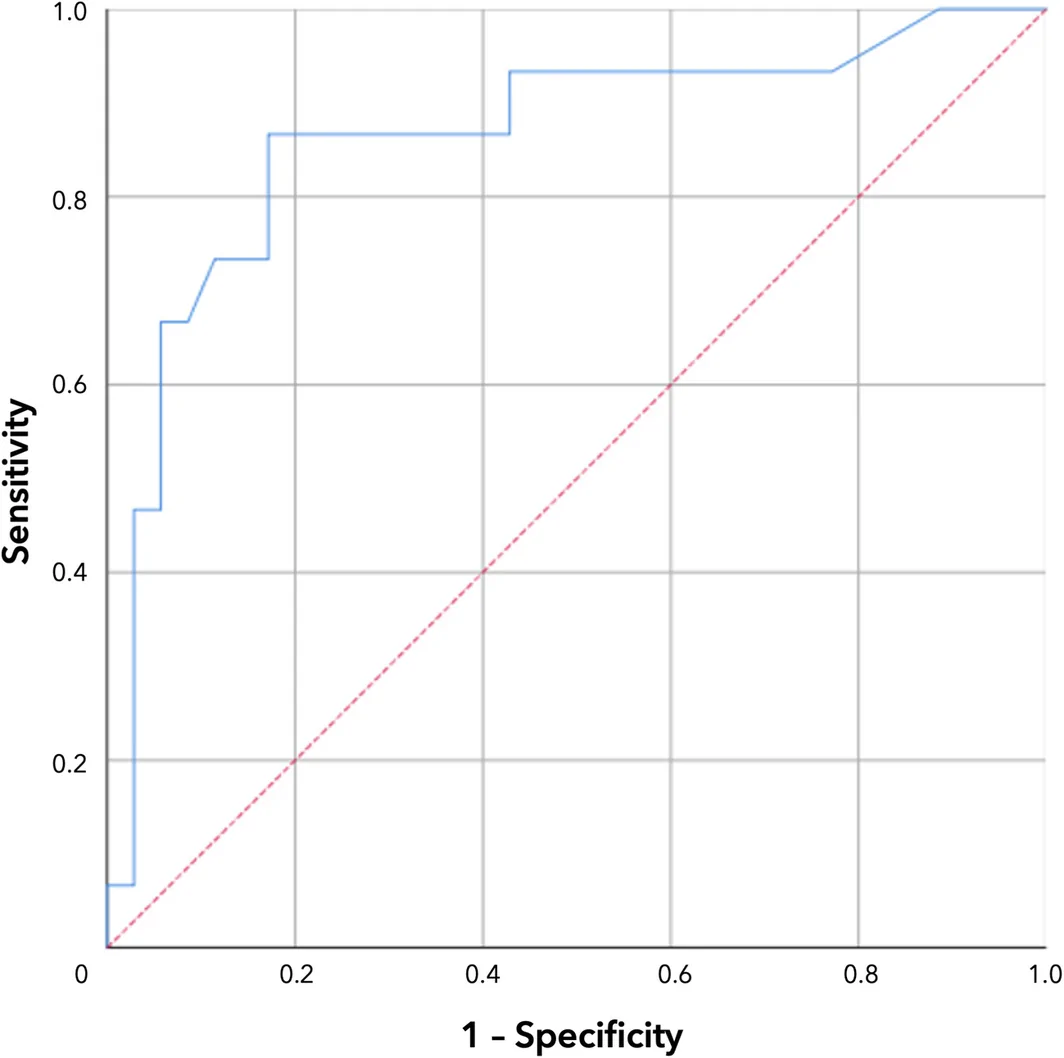
Receiver operating characteristic curve with an area under the curve of 0.86 (95%CI 0.71‐0.95), p < 0.001, classifying a positive Amsterdam Pre‐operative Anxiety and Information Scale (APAIS) status by total Concerns Over Peri‐operative Events (COPE) score (log_10_).

A classification model was used to predict APAIS positivity based on a patient's transformed COPE score. The ROC curve showed good discrimination with an area under the curve of 0.86 (95%CI 0.71–0.95), p < 0.001 (Figure 3). Using a coordinate analysis of this curve, a threshold COPE score of 34 appeared optimal for predicting APAIS positivity, with a sensitivity of 88% and a specificity of 81%.

A COPE score of ≥34 was used to define an individual as having a positive COPE score, identified in 21 (40%) of this study population. On categorical analysis, individuals who had a positive COPE score had significantly higher odds of being APAIS positive (OR 29.0, 95%CI 5.0–158.0, p < 0.001). This association remained robust on subgroup analyses (*p* values ranging from <0.001 to 0.021).

## Discussion

The COPE questionnaire was designed as a 14‐item short‐form assessment of patients' peri‐operative concerns, eliciting responses on a series of five‐point Likert scales. Patients' COPE score correlated well with the APAIS, facilitating the identification of pre‐operative anxiety. Importantly, unlike existing tools, COPE also identifies the individualised likely causes of anxiety. This questionnaire may facilitate a process encouraging clinicians to focus their pre‐operative discussions on the causes of patient anxiety. This is particularly relevant in light of the Montgomery ruling, which highlights the need to tailor consent to include material risks important to the individual [26].

We internally validated the COPE questionnaire as a tool for assessing pre‐operative concerns in our patient cohort. This was performed in a broadly representative population, with diverse demographic and clinical factors (Table 1). However, of note, 87% identified themselves as white, compared to 54% within the 2021 British census [27]. Further, a significant majority had received a previous general anaesthetic, an experience which may have influenced patient perspectives. This may equally explain why more patients felt the anaesthetic had been explained than those who declared to have seen an anaesthetist prior to this surgery.

We established convergent validity by assessing correlation with a related construct, the validated anxiety metric, APAIS. At present, the authors are not aware of any validated tools to directly measure patient concerns. Our results demonstrated COPE scores correlated well with APAIS anxiety scores. Reassuringly, our data, with a mean APAIS anxiety score of 9.0, replicated the mean anxiety score described by Moerman et al. (also 9.0), suggesting similar anxiety levels between the two study populations [14]. Similarly, the number of our patients classified as anxious was also very similar (31% vs. 32%, respectively).

Content validity evaluates how well a tool covers all relevant parts of the construct it aims to measure. We promoted this by consulting an expert panel, using semi‐structured interviews with patients and collecting staff feedback.

Reliability refers to the ability of the tool to give consistent results. We used a measure of internal consistency, Cronbach's α, to assess whether patients responded consistently to different questions measuring the same construct. Our result of 0.93 suggests excellent internal consistency, but also a potential degree of redundancy [28]. We may therefore have classified people as being anxious just as accurately using fewer questions. However, redundancy was expected, given that some questions were left in the questionnaire after the pilot, despite low patient concern ratings, because of their relevance to existing peri‐operative consent practices. Specifically, these included accidental dental damage and having a sore throat. We did not measure test–retest reliability as it was not practical to do this on the day of surgery.

The response process reflects how easily users can engage with the measurement tool. The survey proved feasible within a clinical workflow, with an accessible reading age (12–13 years of age), a relatively fast completion time (median time of 6 min) and a high completion rate. The reading age required to understand the survey was equivalent to a USA seventh‐grade student or a UK reading age of a 12 to 13‐year‐old. This is below the UK average reading age, which equates to a year nine student (13–14 years old) [29]. Although this is an accessible reading age for the majority of adults in the UK, it is above the recommended reading age of 10 to 11 years old for patient medical information; therefore, further language simplification may have been beneficial [29].

Our results showed that patients were most concerned about pain after the operation. This is consistent with several previous studies and implies COPE maintains construct validity relative to the literature [2, 4, 6, 7, 12, 15, 22, 30]. Notably, the next most common concerns were surgical in nature, being major operative complications and an unsuccessful operation. The focus of other studies on solely anaesthetic complications reinforces the utility of our tool [2, 3, 4, 5]. These were survey‐based studies administered in pre‐anaesthesia assessment clinics which, in addition to pain, identified ‘not waking up’, awareness and needles as common fears. It is possible patient concerns dynamically change as the date of surgery approaches, a concept which needs to be addressed in future studies. Our results did not demonstrate any difference in COPE and APAIS scores between subgroups based on age, gender and ASA physical status. This was perhaps surprising because anxiety has previously been associated with sex and age [1, 2, 3, 6, 8, 9, 17, 22, 31]. Similarly, COPE or APAIS were not correlated with self‐declared health score, mental health history, nor having had a previous general anaesthetic. However, given that this project was not designed to assess inter‐group differences, it likely lacked sufficient power to detect such differences.

The COPE questionnaire produces a quantifiable score which could be used to risk‐stratify patients for peri‐operative anxiety, along with identifying specific concerns. A threshold COPE score of 34 was found to predict anxiety with high sensitivity (88%) and specificity (81%). It would classify 40% of our cohort as having a positive COPE score. It favoured a higher sensitivity, such that fewer patients are missed at risk of pre‐operative anxiety. At this level, there is a strong correlation between patients' having a positive COPE and APAIS score, albeit with wide confidence intervals driven by the small sample size and very low number of false negatives. A higher threshold of 40 would favour specificity (86%), at a cost to sensitivity (75%). The COPE score facilitates future studies intended to assess therapeutic interventions targeting patient experience, peri‐operative anxiety and quality of recovery. Validation of the COPE questionnaire in the early pre‐operative phase, such as in pre‐assessment clinics, will be a necessary first step. Addressing concerns in the outpatient setting is preferable, as patients are less likely distracted by the symptoms of anxiety felt on the day of surgery. This is analogous to consent, where some suggest that on‐the‐day consent has practical and ethical concerns: with insufficient time to discuss serious and complex risks, and that if rushed, gives concerns of coercion or exacerbating negative symptoms [32, 33, 34, 35, 36]. Integrating the COPE questionnaire into the pre‐operative patient journey may be an effective way of both addressing concerns, ameliorating anxiety and improving consenting practice.

This study has several limitations. The questionnaire is not yet generalisable, requiring external validation. Data were collected from a single centre, with a small sample size. As the survey was a service evaluation exercise administered on the day of surgery, no changes to care could be made based on the COPE questionnaire results. Thus, we cannot associate the administration of COPE with any change in patient outcomes, and a further research study is now required. We did not include emergency surgery due to the practicalities of administering the survey pre‐operatively to patients requiring urgent surgery; therefore, it is unclear how the survey would perform in this setting. Similarly, obstetric patients were excluded since they are likely to have additional unmeasured concerns hampering applicability.

## Conclusions

In summary, we developed a novel questionnaire to assess patient concerns before surgery. It performed well according to measures of validity, reliability and patient acceptability. An individual's COPE score appears to accurately classify pre‐operative anxiety status, while also highlighting underlying concerns to inform consent. Future research should focus on ensuring generalisability of the survey and using it to introduce targeted therapies to help reduce pre‐operative anxiety.

## Code availability statement

No computational code was generated during this study.

## Supporting information


**Appendix S1.** The Concerns Over Peri‐operative Events (COPE) survey, capturing patient demographic data, details of their surgical operation, along with their peri‐operative concerns.
**Appendix S2.** The surgical and process characteristics of the patients (n = 52). Surgical severity grading was derived from the National Institute for Health and Care Excellence (NICE) pre‐operative testing guidelines [NG45, 2016]. Categorical variables rounded to integers. TAVI, transcatheter aortic valve implantation; VATS, video‐assisted thoracoscopic surgery; AV, arteriovenous.

## Data Availability

The datasets used and analysed during the current study are available from the corresponding author on reasonable request.

## References

[1] Wetsch WA , Pircher I , Lederer W , et al. Preoperative stress and anxiety in day‐care patients and inpatients undergoing fast‐track surgery. Br J Anaesth 2009; 103: 199–205. 10.1093/bja/aep136. 19483203

[2] Mavridou P , Dimitriou V , Manataki A , Arnaoutoglou E , Papadopoulos G . Patient's anxiety and fear of anesthesia: effect of gender, age, education, and previous experience of anesthesia. A survey of 400 patients. J Anesth 2013; 27: 104–108. 10.1007/s00540-012-1460-0. 22864564

[3] Ruhaiyem ME , Alshehri AA , Saade M , Shoabi TA , Zahoor H , Tawfeeq NA . Fear of going under general anesthesia: a cross‐sectional study. Saudi J Anaesth 2016; 10: 317–321. 10.4103/1658-354X.179094. 27375388 PMC4916817

[4] Bheemanna NK , Channaiah SRD , Gowda PKV , Shanmugham VH , Chanappa NM . Fears and perceptions associated with regional anesthesia: a study from a tertiary care hospital in South India. Anesth Essays Res 2017; 11: 483–488. 10.4103/aer.AER_51_17. 28663646 PMC5490094

[5] Bedaso A , Mekonnen N , Duko B . Prevalence and factors associated with preoperative anxiety among patients undergoing surgery in low‐income and middle‐income countries: a systematic review and meta‐analysis. BMJ Open 2022; 12: e058187. 10.1136/bmjopen-2021-058187. PMC8919464 35277412

[6] Walker EMK , Bell M , Cook TM , et al. Patient reported outcome of adult perioperative anaesthesia in the United Kingdom: a cross‐sectional observational study. Br J Anaesth 2016; 117: 758–766. 10.1093/bja/aew381. 27956674

[7] Kil HK , Kim WO , Chung WY , Kim GH , Seo H , Hong JY . Preoperative anxiety and pain sensitivity are independent predictors of propofol and sevoflurane requirements in general anaesthesia. Br J Anaesth 2012; 108: 119–125. 10.1093/bja/aer305. 22084330

[8] Van den Bosch JE , Moons KG , Bonsel GJ , Kalkman CJ . Does measurement of preoperative anxiety have added value for predicting postoperative nausea and vomiting? Anesth Analg 2005; 100: 1525–1532. 10.1213/01.ANE.0000149325.20542.D4. 15845719

[9] Myles PS , Williams DL , Hendrata M , Anderson H , Weeks AM . Patient satisfaction after anaesthesia and surgery: results of a prospective survey of 10,811 patients. Br J Anaesth 2000; 84: 6–10. 10.1093/oxfordjournals.bja.a013383. 10740539

[10] Celik F , Edipoglu IS . Evaluation of preoperative anxiety and fear of anesthesia using APAIS score. Eur J Med Res 2018; 23: 41. 10.1186/s40001-018-0339-4. 30205837 PMC6131845

[11] Eberhart L , Aust H , Schuster M , et al. Preoperative anxiety in adults ‐ a cross‐sectional study on specific fears and risk factors. BMC Psychiatry 2020; 20: 140. 10.1186/s12888-020-02552-w. 32228525 PMC7106568

[12] Shafer A , Fish MP , Gregg KM , Seavello J , Kosek P . Preoperative anxiety and fear: a comparison of assessments by patients and anesthesia and surgery residents. Anesth Analg 1996; 83: 1285–1291. 10.1097/00000539-199612000-00027. 8942601

[13] Kindler CH , Harms C , Amsler F , Ihde‐Scholl T , Scheidegger D . The visual analog scale allows effective measurement of preoperative anxiety and detection of patients' anesthetic concerns. Anesth Analg 2000; 90: 706–712. 10.1097/00000539-200003000-00036. 10702461

[14] Moerman N , van Dam FS , Muller MJ , Oosting H . The Amsterdam preoperative anxiety and information scale (APAIS). Anesth Analg 1996; 82: 445–451. 10.1097/00000539-199603000-00002. 8623940

[15] Wu H , Zhao X , Chu S , et al. Validation of the Chinese version of the Amsterdam preoperative anxiety and information scale (APAIS). Health Qual Life Outcomes 2020; 18: 66. 10.1186/s12955-020-01294-3. 32160883 PMC7065348

[16] Vergara‐Romero M , Morales‐Asencio JM , Morales‐Fernandez A , Canca‐Sanchez JC , Rivas‐Ruiz F , Reinaldo‐Lapuerta JA . Validation of the Spanish version of the Amsterdam preoperative anxiety and information scale (APAIS). Health Qual Life Outcomes 2017; 15: 120. 10.1186/s12955-017-0695-8. 28592310 PMC5463326

[17] Berth H , Petrowski K , Balck F . The Amsterdam Preoperative Anxiety and Information Scale (APAIS) – the first trial of a German version. Psychosoc Med 2007 Feb 20; 4: Doc01. 19742298 PMC2736533

[18] Yentis SM , Hartle AJ , Barker IR , et al. AAGBI: consent for anaesthesia 2017: Association of Anaesthetists of Great Britain and Ireland. Anaesthesia 2017; 72: 93–105. 10.1111/anae.13762. 27988961 PMC6680217

[19] Health Research Authority . UK Policy Framework for Health and Social Care Research. 2023. https://www.hra.nhs.uk/planning‐and‐improving‐research/policies‐standards‐legislation/uk‐policy‐framework‐health‐social‐care‐research/.

[20] Boker A , Brownell L , Donen N . The Amsterdam preoperative anxiety and information scale provides a simple and reliable measure of preoperative anxiety. Can J Anaesth 2002; 49: 792–798. 10.1007/BF03017410. 12374706

[21] Klafta JM , Roizen MF . Current understanding of patients' attitudes toward and preparation for anesthesia: a review. Anesth Analg 1996; 83: 1314–1321. 10.1097/00000539-199612000-00031. 8942605

[22] Powell R , Scott NW , Manyande A , et al. Psychological preparation and postoperative outcomes for adults undergoing surgery under general anaesthesia. Cochrane Database Syst Rev 2016; 2016: CD008646. 10.1002/14651858.CD008646.pub2. 27228096 PMC8687603

[23] National Insitute for Care Excellence . Routine preoperative tests for elective surgery. 2016. https://www.nice.org.uk/guidance/ng45. 41643037

[24] Laerd Statistics . Principal Components Analysis (PCA) using SPSS Statistics. https://statistics.laerd.com/spss‐tutorials/principal‐components‐analysis‐pca‐using‐spss‐statistics.php.

[25] Terwee CB , Bot SD , de Boer MR , et al. Quality criteria were proposed for measurement properties of health status questionnaires. J Clin Epidemiol 2007; 60: 34–42. 10.1016/j.jclinepi.2006.03.012. 17161752

[26] The General Medical Council . Decision making and consent. 2020. https://www.gmc‐uk.org/ethical‐guidance/ethical‐guidance‐for‐doctors/decision‐making‐and‐consent.

[27] Office of National Statistics . Census. 2021. https://www.ons.gov.uk/census.

[28] McDowell I , Newell C . Measuring health: a guide to rating scales and questionnaires. Vol xix. 2nd ed. New York: Oxford University Press, 1996. 523.

[29] Graham C , Reynard JM , Turney BW . Consent information leaflets—readable or unreadable? J Clin Urol 2015; 8: 177–182. 10.1177/2051415814555947. 27867520 PMC5113811

[30] Barnett SF , Alagar RK , Grocott MP , Giannaris S , Dick JR , Moonesinghe SR . Patient‐satisfaction measures in anesthesia: qualitative systematic review. Anesthesiology 2013; 119: 452–478. 10.1097/ALN.0b013e3182976014. 23669268

[31] Laufenberg‐Feldmann R , Kappis B . Assessing preoperative anxiety using a questionnaire and clinical rating: a prospective observational study. Eur J Anaesthesiol 2013; 30: 758–763. 10.1097/EJA.0b013e3283631751. 23787971

[32] Ricketts D , Roper T , Rogers B , Phadnis J , Elsayed S , Sokol D . Informed consent: the view from the trenches. Ann R Coll Surg Engl 2019; 101: 44–49. 10.1308/rcsann.2018.0140. 30286630 PMC6303806

[33] O'Neill O . Some limits of informed consent. J Med Ethics 2003; 29: 4–7. 10.1136/jme.29.1.4. 12569185 PMC1733683

[34] Krauss BS . “this may hurt”: predictions in procedural disclosure may do harm. BMJ 2015; 350: h649. 10.1136/bmj.h649. 25663166 PMC4707520

[35] Cyna AM , Simmons SW . Guidelines on informed consent in anaesthesia: unrealistic, unethical, untenable. Br J Anaesth 2017; 119: 1086–1089. 10.1093/bja/aex347. 29028983

[36] Jovaisa T , Norkiene I , Karjagin J , et al. Are we meeting the current standards of consent for anesthesia? An international survey of clinical practice. Med Sci Monit 2020; 26: e925905. 10.12659/MSM.925905. 33012779 PMC7545782

