# Supplementary material for: Development of a survey evaluating patient Concerns Over Peri‐operative Events (COPE)
Source: Anaesth Rep. 2026 Sep 2;14(2):e70098. doi: 10.1002/anr3.70098 (PMC13537895; doi:10.1002/anr3.70098)
Supplement: Supplementary file 1 [file ANR3-14-e70098-s001.docx]

**Appendices**

**Appendix 1**. The Concerns Over Peri-operative Events (COPE) survey, capturing patient demographic data, details of their surgical operation, along with their peri-operative concerns.

| **Characteristic** | **Count** | **Percentage** |
| --- | --- | --- |
| **Day case status** |  |  |
| Inpatient | 32 | 62% |
| Day case | 18 | 35% |
| No data | 2 | 4% |
| **Primary anaesthetic mode** |  |  |
| General anaesthesia | 46 | 88% |
| Regional | 3 | 6% |
| Sedation | 2 | 4% |
| No data | 1 | 2% |
| **Session start time** |  |  |
| Morning | 27 | 52% |
| Afternoon | 19 | 37% |
| No data | 6 | 12% |
| **List position** |  |  |
| 1st on list | 11 | 21% |
| 2nd on list | 20 | 39% |
| 3rd on list | 11 | 21% |
| No data | 10 | 19% |
| **Surgical severity** |  |  |
| Major | 18 | 35% |
| Moderate | 16 | 31% |
| Minor | 6 | 12% |
| Complex major | 8 | 15% |
| No data | 4 | 8% |
| **Specialty & key procedures** |  |  |
| **Urology** *(cysto/urethroscopy, cystoprostatectomy)* | 10 | 19% |
| **Orthopaedics** *(shoulder, hip & knee replacement)* | 9 | 17% |
| **Gastrointestinal** *(hemicolectomy, cholecystectomy)* | 8 | 15% |
| **Cardiac** *(TAVI, aortic valve replacement)* | 7 | 14% |
| **Ear, Nose & Throat (ENT)** *(panendoscopy, parotidectomy)* | 3 | 6% |
| **Gynaecology** *(hysterectomy, ovarian cystectomy)* | 3 | 6% |
| **Thoracic** *(VATS, bronchoscopy)* | 3 | 6% |
| **Transplant** *(AV fistula formation)* | 2 | 4% |
| **Breast** *(mastectomy, implant exchange)* | 2 | 4% |
| **Pain** *(neurostimulator insertion)* | 2 | 4% |
| **Maxillofacial** *(tracheostomy, neck dissection)* | 2 | 4% |
| **Vascular** *(carotid endarterectomy)* | 1 | 2% |

**Appendix 2**. The surgical and process characteristics of the patients (n = 52). Surgical severity grading was derived from the National Institute for Health and Care Excellence (NICE) pre-operative testing guidelines [NG45, 2016]. Categorical variables rounded to integers. TAVI, Transcatheter Aortic Valve Implantation; VATS, Video-Assisted Thoracoscopic Surgery; AV, arteriovenous.
